# Crystal structure of 1-benzyl­sulfonyl-1,2,3,4-tetra­hydro­quinoline

**DOI:** 10.1107/S2056989015004727

**Published:** 2015-03-21

**Authors:** S. Jeyaseelan, B. R. Sowmya, G. Venkateshappa, P. Raghavendra Kumar, B. S. Palakshamurthy

**Affiliations:** a Department of Physics, St Philomena’s College (Autonomous), Mysore, Karnataka 570 015, India; b Department of Studies and Research in Physics, U.C.S., Tumkur University, Tumkur, Karnataka 572 103, India; c Department of Chemistry, Tumkur University, Tumkur, Karnataka 572 103, India; d Department of Studies and Research in Chemistry, Tumkur University, Tumkur University, Tumkur, Karnataka 572 103, India

**Keywords:** crystal structure, 1,2,3,4-tetra­hydro­quinoline, weak C—H⋯O inter­actions

## Abstract

In the title compound, C_16_H_17_NO_2_S, the heterocyclic ring adopts a half-chair conformation and the bond-angle sum at the N atom is 354.6°. The dihedral angle between the planes of the aromatic rings is 74.15 (10)°. In the crystal, mol­ecules are linked by weak C—H⋯O hydrogen bonds, generating *C*(8) and *C*(4) chains propagating along [100] and [010], respectively, which together generate (001) sheets.

## Related literature   

For the biological properties of 1,2,3,4-tetra­hydro­quinoline derivatives, see: Bendale *et al.* (2007 ▸); Singer *et al.* (2005 ▸). For related structures, see: Jeyaseelan *et al.* (2014 ▸, 2015 ▸).
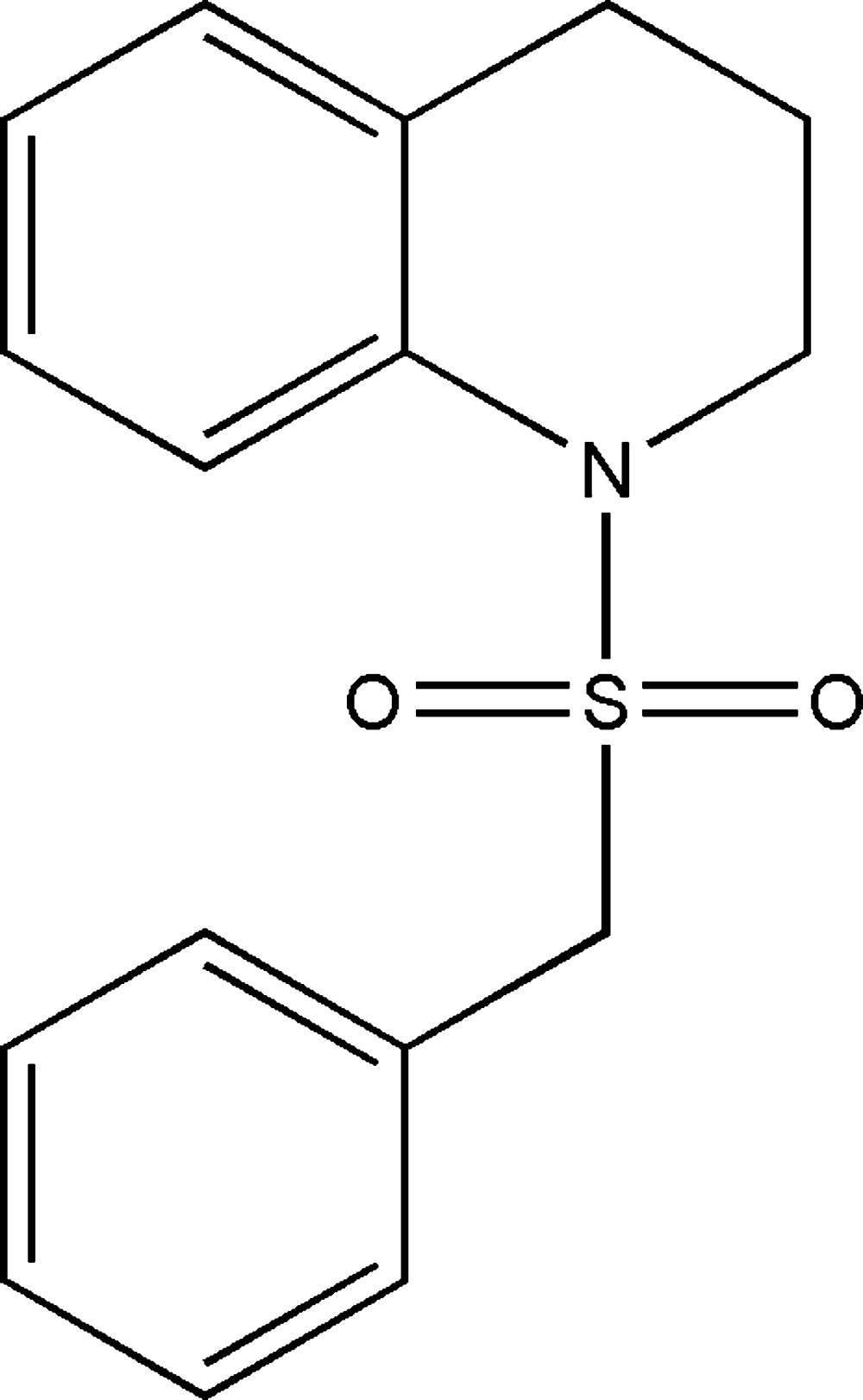


## Experimental   

### Crystal data  

C_16_H_17_NO_2_S
*M* *_r_* = 287.36
Monoclinic, 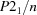

*a* = 13.5690 (5) Å
*b* = 6.7128 (2) Å
*c* = 16.8317 (6) Å
β = 110.243 (1)°
*V* = 1438.44 (9) Å^3^
*Z* = 4
Mo *K*α radiation
μ = 0.23 mm^−1^
*T* = 295 K
0.24 × 0.20 × 0.18 mm

### Data collection  

Bruker APEXII CCD diffractometer
Absorption correction: multi-scan (*SADABS*; Bruker, 2013 ▸) *T*_min_ = 0.947, *T*_max_ = 0.960
19601 measured reflections
2529 independent reflections
2264 reflections with *I* > 2σ(*I*)
*R*_int_ = 0.051

### Refinement  

*R*[*F* ^2^ > 2σ(*F* ^2^)] = 0.036
*wR*(*F* ^2^) = 0.101
*S* = 1.08
2529 reflections
181 parameters
H-atom parameters constrained
Δρ_max_ = 0.15 e Å^−3^
Δρ_min_ = −0.38 e Å^−3^

### 

Data collection: *APEX2* (Bruker, 2013 ▸); cell refinement: *SAINT* (Bruker, 2013 ▸); data reduction: *SAINT*; program(s) used to solve structure: *SHELXS97* (Sheldrick, 2008 ▸); program(s) used to refine structure: *SHELXL2014* (Sheldrick, 2015 ▸); molecular graphics: *Mercury* (Macrae *et al.*, 2008 ▸); software used to prepare material for publication: *SHELXL2014*.

## Supplementary Material

Crystal structure: contains datablock(s) I. DOI: 10.1107/S2056989015004727/hb7377sup1.cif

Structure factors: contains datablock(s) I. DOI: 10.1107/S2056989015004727/hb7377Isup2.hkl

Supporting information file. DOI: 10.1107/S2056989015004727/hb7377Isup3.cml

**Fig. 1.**
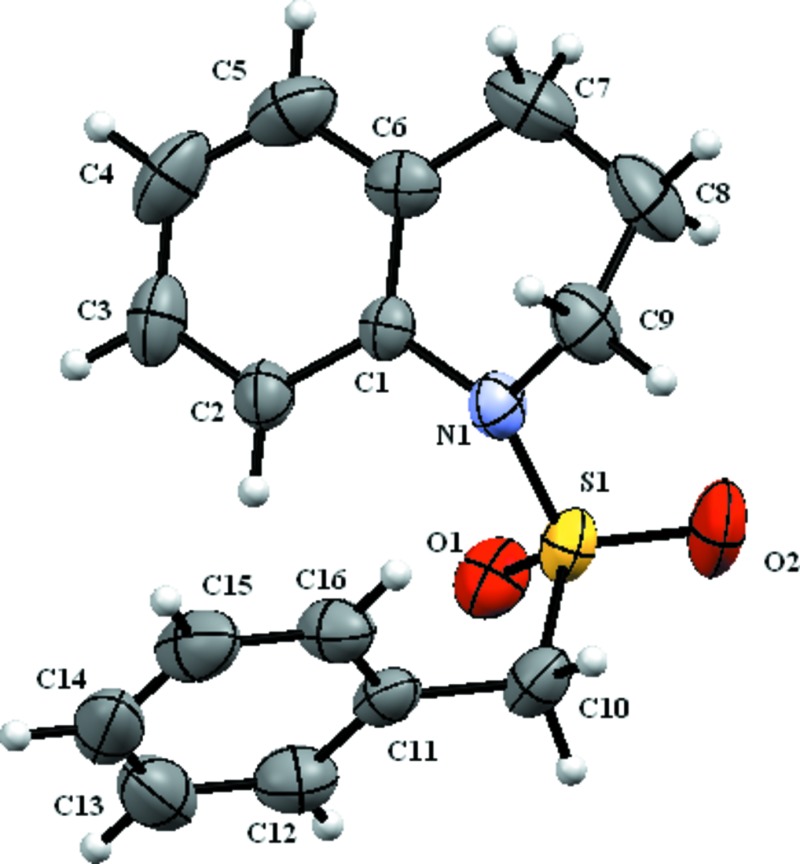
The molecular structure of the title compound, showing displacement ellipsoids drawn at the 50% probability level.

**Fig. 2.**
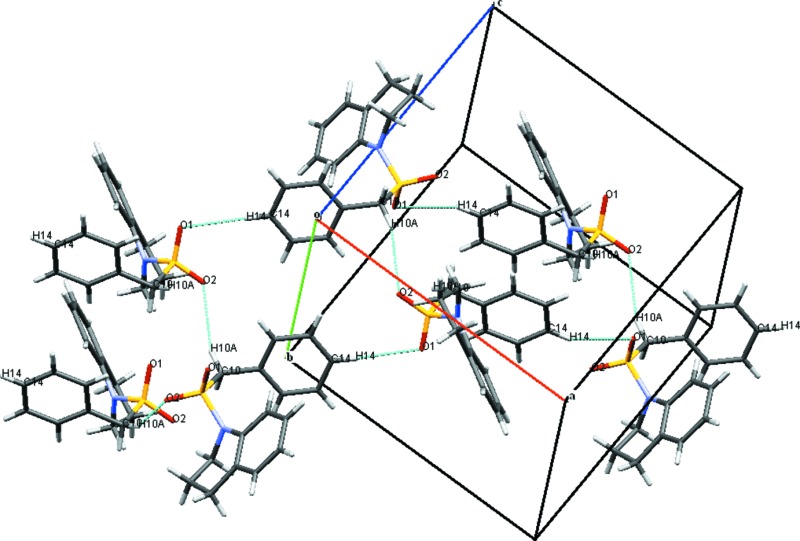
The molecular packing of the title compound, dashed lines indicates the C—H···O weak hydrogen bonds in the ab plane.

. DOI: 10.1107/S2056989015004727/hb7377fig1.tif
The mol­ecular structure of the title compound, showing displacement ellipsoids drawn at the 50% probability level.

ab . DOI: 10.1107/S2056989015004727/hb7377fig2.tif
The mol­ecular packing of the title compound, dashed lines indicates the C—H⋯O weak hydrogen bonds in the *ab* plane.

CCDC reference: 1052632

Additional supporting information:  crystallographic information; 3D view; checkCIF report

## Figures and Tables

**Table 1:** Hydrogen-bond geometry (, )

*D*H*A*	*D*H	H*A*	*D* *A*	*D*H*A*
C14H14O1^i^	0.93	2.69	3.573(2)	158
C10H10*A*O2^ii^	0.97	2.68	3.575(2)	153

Symmetry codes: (i) 
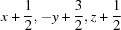
; (ii) 
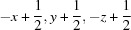
.

## supplementary crystallographic information

### S1. Introduction

Heterocyclic compounds of 1,2,3,4-tetra­hydro­quinoline derivatives play important role in synthesize anti­malarial (Bendale *et al.*, 2007), anti­psychotic (Singer *et al.*, 2005) drugs. Keeping this in mind we have synthised a series of 1,2,3,4-tetra­hydro­quinoline with derivatives of Sulfonyl chlorides they exhibit a few pharmacological activities (our unpublished data). As a part of our study we have undertaken crystal structure determination of the title compound(I) and the results are compared with crystal structure of 1- tosyl-1,2,3,4-tetra­hydro­quinoline­(II) and 1-methane­sulfonyl-1,2,3,4-tetra­hydro­quinoline­(III) (Jeyaseelan *et al.*, 2014a & 2014b).

### S2. Structural commentary

The molecular structure of the title compound (I) is shown in Fig. 1. In all the compounds (I),(II) and (III), the C1/C6–C9/N1 rings are in a half-chair conformation, but the bond-angle sum at the N atom in the compound (I), (II) and (III) are 354.61°, 347.9° and 350.2°, respectively.

The crystal structure of (I) features C14–H14···O1 weak hydrogen bonds generating C(8) chain along [100] and C10–H10A···O2 weak hydrogen bonds generating C(4) along [010]: together these generate (001) sheets.

### S3. Experimental

To a stirred solution of 1,2,3,4-tetra­hydro­quinoline (10 mmol) in 30 ml dry methyl­ene dichloride, tri­ethyl­amine (15 mmol) was added at 0–5°C. To this reaction mixture phenyl­methane­sulfonyl chloride (12 mmol) in 10 ml dry di­chloro­methane was added drop wise. After 2h of stirring at 15–20°C, the reaction mixture was washed with 5% Na_2_CO_3_ and brine. The organic phase was dried over Na_2_SO_4_ and then it was concentrated on vacuum to yield titled compound as colourless solid. The crude product was recrystallized from a solvent mixture of ethyl acetate and hexane (1:2) to yield colourless prisms of (I).

### S3.1. Refinement

Crystal data, data collection and structure refinement details are summarized in Table 1. The H atoms were positioned with idealized geometry using a riding model with C—H = 0.93-0.97 Å. All H-atoms were refined with isotropic displacement parameters (set to 1.2-1.5 times of the U eq of the parent atom).

### Crystal data

**Table:**

C_16_H_17_NO_2_S	Prism
*M**_r_* = 287.36	*D*_x_ = 1.327 Mg m^−^^3^
Monoclinic, *P*2_1_/*n*	Melting point: 514 K
Hall symbol: -P 2yn	Mo *K*α radiation, λ = 0.71073 Å
*a* = 13.5690 (5) Å	Cell parameters from 2529 reflections
*b* = 6.7128 (2) Å	θ = 1.7–25°
*c* = 16.8317 (6) Å	µ = 0.23 mm^−^^1^
β = 110.243 (1)°	*T* = 295 K
*V* = 1438.44 (9) Å^3^	Prism, colourless
*Z* = 4	0.24 × 0.20 × 0.18 mm
*F*(000) = 608	

### Data collection

**Table:**

Bruker APEXII CCD diffractometer	2529 independent reflections
Radiation source: fine-focus sealed tube	2264 reflections with *I* > 2σ(*I*)
Graphite monochromator	*R*_int_ = 0.051
Detector resolution: 1.90 pixels mm^-1^	θ_max_ = 25.0°, θ_min_ = 1.7°
phi and ω scans	*h* = −16→16
Absorption correction: multi-scan (*SADABS*; Bruker, 2013)	*k* = −7→7
*T*_min_ = 0.947, *T*_max_ = 0.960	*l* = −19→20
19601 measured reflections	

### Refinement

**Table:**

Refinement on *F*^2^	Primary atom site location: structure-invariant direct methods
Least-squares matrix: full	Secondary atom site location: difference Fourier map
*R*[*F*^2^ > 2σ(*F*^2^)] = 0.036	Hydrogen site location: inferred from neighbouring sites
*wR*(*F*^2^) = 0.101	H-atom parameters constrained
*S* = 1.08	*w* = 1/[σ^2^(*F*_o_^2^) + (0.0563*P*)^2^ + 0.3073*P*] where *P* = (*F*_o_^2^ + 2*F*_c_^2^)/3
2529 reflections	(Δ/σ)_max_ < 0.001
181 parameters	Δρ_max_ = 0.15 e Å^−^^3^
0 restraints	Δρ_min_ = −0.38 e Å^−^^3^
0 constraints	

### Special details

**Table:**

Geometry. All e.s.d.'s (except the e.s.d. in the dihedral angle between two l.s. planes) are estimated using the full covariance matrix. The cell e.s.d.'s are taken into account individually in the estimation of e.s.d.'s in distances, angles and torsion angles; correlations between e.s.d.'s in cell parameters are only used when they are defined by crystal symmetry. An approximate (isotropic) treatment of cell e.s.d.'s is used for estimating e.s.d.'s involving l.s. planes.

### Fractional atomic coordinates and isotropic or equivalent isotropic displacement parameters (Å^2^)

**Table:**

	*x*	*y*	*z*	*U*_iso_*/*U*_eq_	
S1	0.36706 (3)	0.52581 (6)	0.20696 (2)	0.04077 (16)	
N1	0.47088 (9)	0.40403 (18)	0.20485 (8)	0.0377 (3)	
O1	0.37882 (9)	0.73063 (17)	0.18920 (8)	0.0515 (3)	
C11	0.47391 (11)	0.5739 (2)	0.37732 (10)	0.0395 (4)	
C1	0.60202 (12)	0.6674 (2)	0.22242 (10)	0.0419 (4)	
H1	0.5827	0.7225	0.2656	0.050*	
O2	0.27373 (9)	0.4236 (2)	0.15618 (8)	0.0634 (4)	
C6	0.55139 (11)	0.4980 (2)	0.18039 (9)	0.0334 (3)	
C12	0.55291 (13)	0.4389 (3)	0.41491 (11)	0.0488 (4)	
H12	0.5430	0.3051	0.3999	0.059*	
C5	0.58183 (13)	0.4084 (2)	0.11812 (10)	0.0448 (4)	
C10	0.37241 (13)	0.5045 (3)	0.31416 (11)	0.0477 (4)	
H10A	0.3155	0.5818	0.3211	0.057*	
H10B	0.3615	0.3662	0.3257	0.057*	
C9	0.46039 (15)	0.1865 (2)	0.19022 (11)	0.0516 (4)	
H9A	0.4017	0.1368	0.2047	0.062*	
H9B	0.5236	0.1200	0.2260	0.062*	
C16	0.49091 (15)	0.7733 (3)	0.40037 (12)	0.0547 (4)	
H16	0.4387	0.8669	0.3758	0.066*	
C2	0.68073 (13)	0.7538 (3)	0.20020 (13)	0.0555 (5)	
H2	0.7137	0.8689	0.2275	0.067*	
C3	0.71051 (15)	0.6695 (3)	0.13749 (14)	0.0679 (6)	
H3	0.7633	0.7280	0.1221	0.081*	
C13	0.64626 (15)	0.4993 (3)	0.47439 (12)	0.0632 (5)	
H13	0.6984	0.4061	0.4996	0.076*	
C14	0.66280 (15)	0.6951 (4)	0.49666 (12)	0.0646 (6)	
H14	0.7262	0.7354	0.5366	0.078*	
C7	0.53441 (17)	0.2149 (3)	0.07532 (12)	0.0625 (5)	
H7A	0.5113	0.2333	0.0145	0.075*	
H7B	0.5885	0.1131	0.0903	0.075*	
C4	0.66236 (16)	0.4995 (3)	0.09785 (14)	0.0634 (5)	
H4	0.6840	0.4431	0.0562	0.076*	
C8	0.44285 (18)	0.1420 (3)	0.09850 (13)	0.0667 (6)	
H8A	0.4346	−0.0005	0.0888	0.080*	
H8B	0.3789	0.2067	0.0629	0.080*	
C15	0.58539 (18)	0.8325 (3)	0.45973 (13)	0.0654 (5)	
H15	0.5966	0.9662	0.4747	0.079*	

### Atomic displacement parameters (Å^2^)

**Table:**

	*U*^11^	*U*^22^	*U*^33^	*U*^12^	*U*^13^	*U*^23^
S1	0.0284 (2)	0.0475 (3)	0.0419 (3)	−0.00260 (14)	0.00645 (17)	0.00823 (16)
N1	0.0394 (7)	0.0304 (6)	0.0437 (7)	−0.0068 (5)	0.0147 (5)	−0.0008 (5)
O1	0.0455 (7)	0.0438 (7)	0.0627 (7)	0.0085 (5)	0.0155 (6)	0.0154 (5)
C11	0.0358 (8)	0.0506 (9)	0.0373 (8)	0.0036 (7)	0.0193 (6)	0.0022 (7)
C1	0.0376 (8)	0.0386 (8)	0.0478 (9)	−0.0041 (6)	0.0127 (7)	−0.0021 (7)
O2	0.0352 (6)	0.0850 (9)	0.0578 (8)	−0.0170 (6)	0.0004 (5)	0.0074 (7)
C6	0.0308 (7)	0.0326 (7)	0.0349 (8)	−0.0002 (5)	0.0088 (6)	0.0035 (6)
C12	0.0503 (10)	0.0531 (10)	0.0431 (9)	0.0111 (8)	0.0165 (8)	−0.0011 (7)
C5	0.0502 (9)	0.0451 (9)	0.0392 (8)	0.0065 (7)	0.0157 (7)	0.0047 (7)
C10	0.0347 (8)	0.0621 (10)	0.0496 (10)	0.0006 (7)	0.0187 (7)	0.0069 (8)
C9	0.0659 (11)	0.0311 (8)	0.0573 (10)	−0.0103 (7)	0.0205 (9)	0.0010 (7)
C16	0.0605 (11)	0.0515 (10)	0.0575 (11)	0.0093 (8)	0.0272 (9)	0.0028 (8)
C2	0.0405 (9)	0.0500 (10)	0.0697 (12)	−0.0115 (7)	0.0108 (8)	0.0085 (9)
C3	0.0477 (10)	0.0809 (14)	0.0836 (14)	−0.0053 (10)	0.0336 (10)	0.0238 (12)
C13	0.0463 (10)	0.0953 (16)	0.0442 (10)	0.0197 (10)	0.0107 (8)	0.0000 (10)
C14	0.0509 (10)	0.1025 (17)	0.0424 (10)	−0.0162 (11)	0.0185 (8)	−0.0123 (10)
C7	0.0889 (14)	0.0505 (10)	0.0481 (10)	0.0043 (10)	0.0236 (10)	−0.0111 (8)
C4	0.0630 (12)	0.0794 (14)	0.0605 (12)	0.0072 (10)	0.0375 (10)	0.0079 (10)
C8	0.0895 (15)	0.0432 (10)	0.0611 (12)	−0.0192 (10)	0.0180 (10)	−0.0149 (9)
C15	0.0842 (14)	0.0607 (12)	0.0607 (12)	−0.0209 (11)	0.0368 (11)	−0.0156 (10)

### Geometric parameters (Å, º)

**Table:**

S1—O1	1.4277 (12)	C9—H9A	0.9700
S1—O2	1.4341 (12)	C9—H9B	0.9700
S1—N1	1.6397 (13)	C16—C15	1.383 (3)
S1—C10	1.7863 (18)	C16—H16	0.9300
N1—C6	1.4397 (18)	C2—C3	1.376 (3)
N1—C9	1.4794 (19)	C2—H2	0.9300
C11—C12	1.379 (2)	C3—C4	1.368 (3)
C11—C16	1.390 (2)	C3—H3	0.9300
C11—C10	1.494 (2)	C13—C14	1.364 (3)
C1—C2	1.376 (2)	C13—H13	0.9300
C1—C6	1.389 (2)	C14—C15	1.374 (3)
C1—H1	0.9300	C14—H14	0.9300
C6—C5	1.389 (2)	C7—C8	1.507 (3)
C12—C13	1.376 (3)	C7—H7A	0.9700
C12—H12	0.9300	C7—H7B	0.9700
C5—C4	1.394 (3)	C4—H4	0.9300
C5—C7	1.517 (2)	C8—H8A	0.9700
C10—H10A	0.9700	C8—H8B	0.9700
C10—H10B	0.9700	C15—H15	0.9300
C9—C8	1.508 (3)		
			
O1—S1—O2	118.41 (8)	H9A—C9—H9B	108.2
O1—S1—N1	108.44 (7)	C15—C16—C11	120.08 (18)
O2—S1—N1	109.67 (8)	C15—C16—H16	120.0
O1—S1—C10	108.68 (8)	C11—C16—H16	120.0
O2—S1—C10	106.42 (8)	C1—C2—C3	119.78 (17)
N1—S1—C10	104.30 (7)	C1—C2—H2	120.1
C6—N1—C9	115.08 (12)	C3—C2—H2	120.1
C6—N1—S1	122.03 (10)	C4—C3—C2	119.99 (17)
C9—N1—S1	117.50 (10)	C4—C3—H3	120.0
C12—C11—C16	118.48 (16)	C2—C3—H3	120.0
C12—C11—C10	120.05 (15)	C14—C13—C12	120.44 (18)
C16—C11—C10	121.46 (15)	C14—C13—H13	119.8
C2—C1—C6	120.02 (16)	C12—C13—H13	119.8
C2—C1—H1	120.0	C13—C14—C15	119.68 (18)
C6—C1—H1	120.0	C13—C14—H14	120.2
C1—C6—C5	120.99 (14)	C15—C14—H14	120.2
C1—C6—N1	120.25 (13)	C8—C7—C5	114.05 (15)
C5—C6—N1	118.62 (13)	C8—C7—H7A	108.7
C13—C12—C11	120.92 (17)	C5—C7—H7A	108.7
C13—C12—H12	119.5	C8—C7—H7B	108.7
C11—C12—H12	119.5	C5—C7—H7B	108.7
C6—C5—C4	117.24 (16)	H7A—C7—H7B	107.6
C6—C5—C7	122.76 (15)	C3—C4—C5	121.93 (18)
C4—C5—C7	119.95 (16)	C3—C4—H4	119.0
C11—C10—S1	113.53 (11)	C5—C4—H4	119.0
C11—C10—H10A	108.9	C7—C8—C9	110.39 (15)
S1—C10—H10A	108.9	C7—C8—H8A	109.6
C11—C10—H10B	108.9	C9—C8—H8A	109.6
S1—C10—H10B	108.9	C7—C8—H8B	109.6
H10A—C10—H10B	107.7	C9—C8—H8B	109.6
N1—C9—C8	109.76 (14)	H8A—C8—H8B	108.1
N1—C9—H9A	109.7	C14—C15—C16	120.39 (19)
C8—C9—H9A	109.7	C14—C15—H15	119.8
N1—C9—H9B	109.7	C16—C15—H15	119.8
C8—C9—H9B	109.7		

### Hydrogen-bond geometry (Å, º)

**Table:**

*D*—H···*A*	*D*—H	H···*A*	*D*···*A*	*D*—H···*A*
C14—H14···O1^i^	0.93	2.69	3.573 (2)	158
C10—H10*A*···O2^ii^	0.97	2.68	3.575 (2)	153

Symmetry codes: (i) *x*+1/2, −*y*+3/2, *z*+1/2; (ii) −*x*+1/2, *y*+1/2, −*z*+1/2.
