# Supplementary figures and images for: Crystal structure of 1-benzyl­sulfonyl-1,2,3,4-tetra­hydro­quinoline
Source: Acta Crystallogr E Crystallogr Commun. 2015 Mar 21;71(Pt 4):o249–50. doi: 10.1107/S2056989015004727 (PMC4438803; doi:10.1107/S2056989015004727)

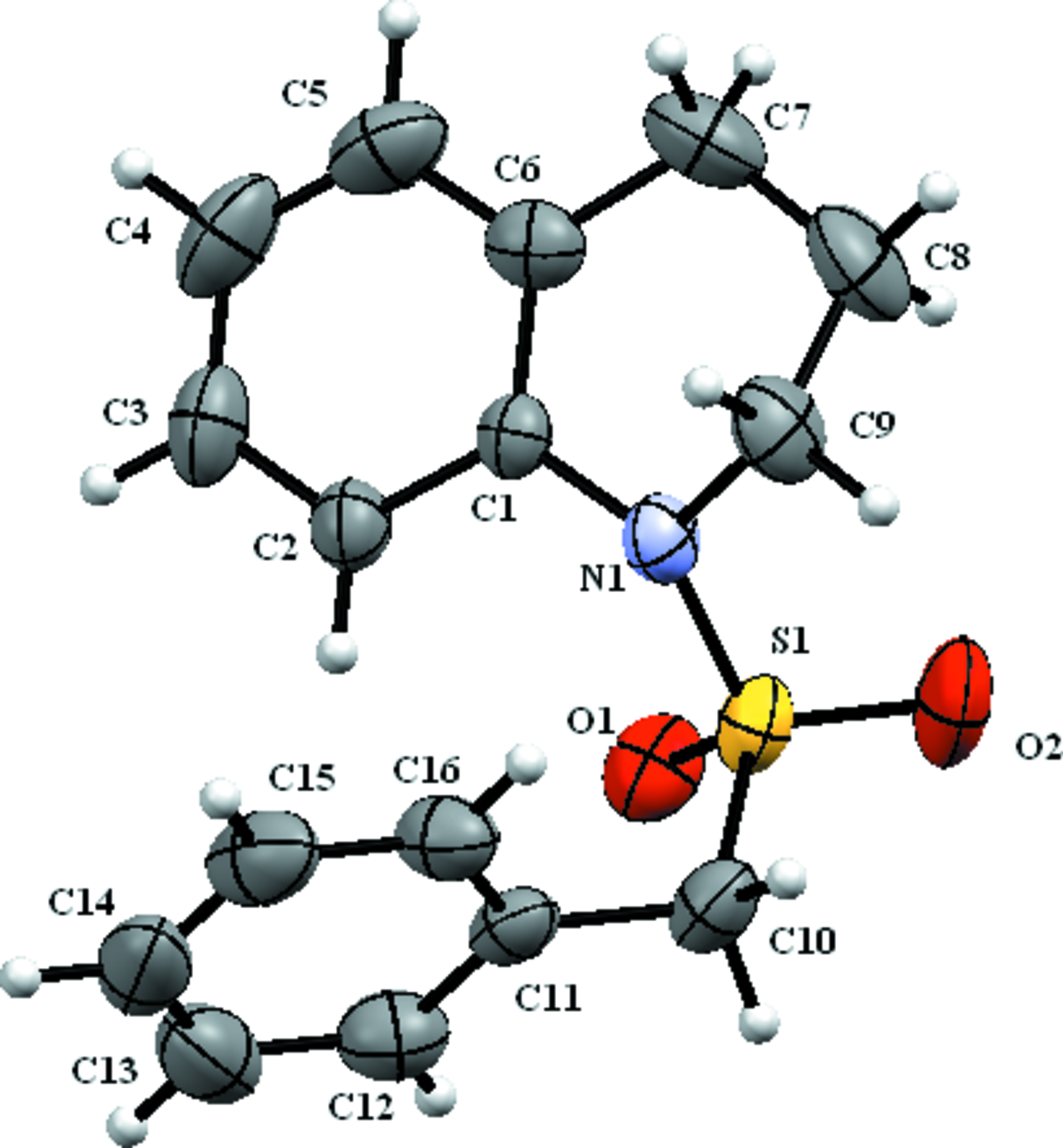

Supplement: Supplementary file 4 [file e-71-0o249-fig1.tif]

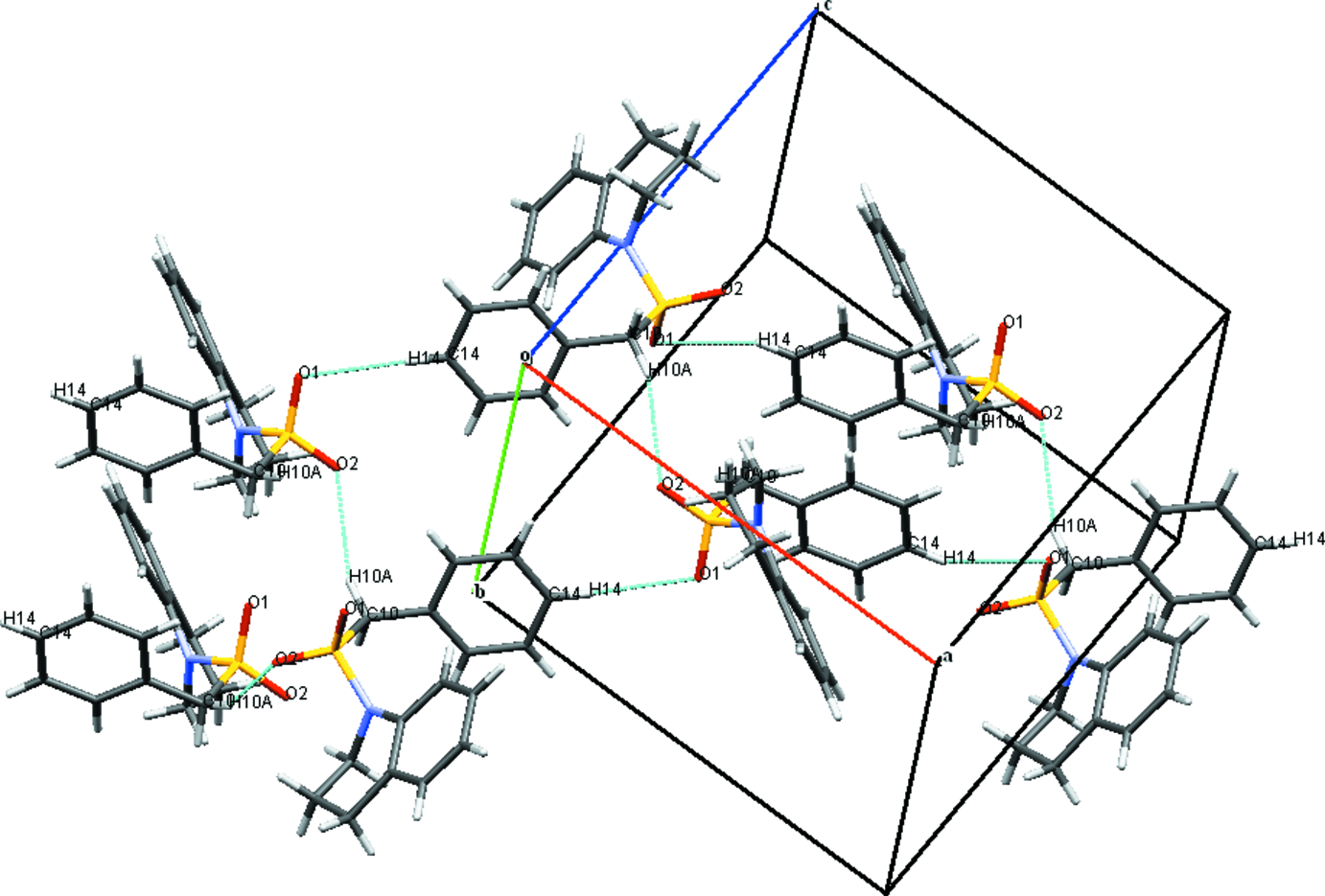

Supplement: Supplementary file 5 [file e-71-0o249-fig2.tif]
